# Low Baseline Interleukin-17A Levels Are Associated with Better Treatment Response at 12 Weeks to Tocilizumab Therapy in Rheumatoid Arthritis Patients

**DOI:** 10.1155/2015/487230

**Published:** 2015-04-02

**Authors:** Sang Jin Lee, Won Park, Sung Hwan Park, Seung-Cheol Shim, Han Joo Baek, Dae-Hyun Yoo, Hyun Ah Kim, Soo Kon Lee, Yun Jong Leee, Young Eun Park, Hoon-Suk Cha, Jin Kyun Park, Eun Young Lee, Eun Bong Lee, Yeong Wook Song

**Affiliations:** ^1^Division of Rheumatology, Seoul National University Hospital, Seoul, Republic of Korea; ^2^Department of Molecular Medicine and Biopharmaceutical Sciences, Graduate School of Convergence Science and Technology and College of Medicine, Medical Research Institute, Seoul National University, Seoul, Republic of Korea; ^3^Division of Rheumatology, Inha University Hospital, Incheon, Republic of Korea; ^4^Division of Rheumatology, The Catholic University of Korea Seoul St. Mary's Hospital, Seoul, Republic of Korea; ^5^Division of Rheumatology, Eulji University Hospital, Daejeon, Republic of Korea; ^6^Division of Rheumatology, Gachon University Gil Medical Center, Incheon, Republic of Korea; ^7^Division of Rheumatology, Hanyang University Medical Center, Seoul, Republic of Korea; ^8^Division of Rheumatology, Hallym University Medical Center, Anyang, Republic of Korea; ^9^Division of Rheumatology, Yonsei University Health System, Seoul, Republic of Korea; ^10^Division of Rheumatology, Seoul National University Bundang Hospital, Seongnam, Republic of Korea; ^11^Division of Rheumatology, Pusan National University Hospital, Busan, Republic of Korea; ^12^Division of Rheumatology, Samsung Medical Center, Seoul, Republic of Korea

## Abstract

T helper 17-related cytokines have been implicated in rheumatoid arthritis (RA) pathogenesis. The study aimed to identify cytokines associated with the treatment response of RA patients to tocilizumab (TCZ), a humanized monoclonal antibody against the interleukin- (IL-) 6 receptor. As an independent substudy of the 24-week, randomized, double-blinded CWP-TCZ301 trial of TCZ in RA patients with an inadequate response to disease-modifying antirheumatic drugs, serum levels of cytokines including tumor necrosis factor-alpha, IL-17A, IL-21, IL-23, IL-6, and soluble IL-6 receptor were measured. Baseline IL-17A levels were significantly lower in RA patients who achieved disease activity score 28 (DAS28) remission at 12 weeks of TCZ treatment, compared to patients not in remission. Patients were stratified into IL-17A low group and IL-17A high group. Significantly more patients in the IL-17A low group achieved remission as compared to the IL-17A high group (47.6 versus 17.4%, *P* = 0.032). DAS28 improvement was significantly better in the IL-17A low group than in the IL-17A high group at 12 weeks (*P* = 0.045) and 24 weeks (*P* = 0.046) after adjustment. Other baseline cytokines were not associated with treatment response to TCZ. The data demonstrate that low baseline IL-17A levels are associated with better clinical response to TCZ treatment in RA patients.

## 1. Introduction

Rheumatoid arthritis (RA) is a chronic systemic autoimmune inflammatory disease that is characterized by synovial inflammation and cartilage and bone destruction [1]. Interleukin- (IL-) 6 is implicated in RA pathogenesis [2, 3]. After binding to the IL-6 receptor (IL-6R), IL-6 mediates a systemic inflammatory response at cellular and organ-specific levels inducing acute phase reactant production in liver and a febrile response [4]. Furthermore, in concert with transforming growth factor beta (TGF-*β*), IL-6 promotes naïve T cells to preferentially differentiate into T helper (Th) 17 cells through a sequential engagement of IL-21 and IL-23. In addition, IL-6 inhibits the generation of regulatory T (Treg) cells. As such, IL-6 is involved in activation of both innate and adaptive immune cells and contributes to chronic inflammatory process in RA [5–7].

Tocilizumab (TCZ) is a humanized monoclonal antibody targeting the IL-6R, which blocks both soluble and membrane bound IL-6R. It is effective in RA treatment and frequently leads to disease remission [8–12]. However, as a significant subset of RA patients still do not respond to TCZ, it is of paramount importance to identify those patients who will clinically respond to TCZ before treatment initiation. Accordingly, several biomarkers have been explored as predictors of treatment response. Low baseline levels of serum IL-6 before TCZ treatment have been associated with the good efficacy at 4 weeks [13]. However, the clinical response at 4 weeks of treatment cannot be necessarily extrapolated into a long-term clinical response. Low baseline levels of soluble IL-6R (sIL-6R) may be a better marker for clinical response to TCZ [14].

As IL-6 is involved in Th17 generation, which contributes to RA pathogenesis, we hypothesized that the additional presence of cytokines involved in Th17 generation and activation might be associated with a worse clinical response to TCZ in RA patients.

## 2. Patients and Methods

### 2.1. Study Population

This study was specifically designed as a substudy of the TCZ study (CWP-TCZ301, registered at www.clinicaltrials.gov, NCT01211834), a 24-week, randomized, double-blinded, multicenter trial of TCZ in RA patients with an inadequate response to disease-modifying antirheumatic drugs (DMARDs). Patients were randomly assigned in a 1 : 1 ratio to receive intravenous infusion of TCZ 8 mg/kg or placebo every 4 weeks with stable dose of methotrexate (MTX) and/or other DMARDs. Patients who did not achieve a 20% improvement from baseline in both swollen joints count (SJC) and tender joints count (TJC) at week 16 were offered escape therapy. For inclusion, patients needed to be ≥18 years of age with moderate-to-severe active RA for ≥6 months. RA was diagnosed according to the revised 1987 American College of Rheumatology criteria. Active RA was defined by the presence of a SJC in six or more of 66 joints, TJC in eight or more of 68 joints, and C-reactive protein (CRP) level ≥1 mg/dL or erythrocyte sedimentation rate (ESR) ≥28 mm/h. Enrolled patients received stable dose of MTX with or without other DMARDs (hydroxychloroquine, parenteral gold, sulfasalazine, azathioprine, and leflunomide) for ≥8 weeks prior to study entry. Oral glucocorticoids (up to 10 mg/day prednisone or equivalent) and nonsteroidal anti-inflammatory drugs were allowed if the dose had been stable for ≥4 weeks prior to the randomization. If patients had received any biologic agents previously, those were stopped with appropriate washout periods (≥2 weeks for etanercept, ≥8 weeks for infliximab or adalimumab, ≥12 weeks for abatacept, and ≥36 weeks for rituximab). Forty-eight patients were enrolled in the TCZ arm. Of these, 44 patients who completed the 12-week treatment with TCZ and 12 age- and sex-matched healthy controls were included.

The protocol was approved by institutional review boards and ethics committees of Seoul National University Hospital, Seoul, Republic of Korea (1302-028-463). All patients gave informed consent. The study was conducted in full concordance with the principles of the Declaration of Helsinki and with the laws and the regulations of Korea.

### 2.2. Sample Collection and Assays

All samples were immediately stored at −80°C until cytokine assay. Serum IL-6, sIL-6R, and rheumatoid factor (RF) were assayed using appropriate enzyme linked immunosorbent assays (ELISAs) (IL-6 and sIL-6R, eBioscience, San Diego, CA, USA, and RF, Nittobo, Tokyo, Japan). The levels of TNF-*α*, IL-17A, IL-21, and IL-23 were measured using a multiplex bead immunoassay with Luminex laser based fluorescent analytical test instrumentation (Merck-Millipore, Darmstadt, Germany). All assays were conducted in duplicate.

### 2.3. Statistical Analyses

Mann-Whitney* U* test was performed for group comparisons and Fisher's exact test was used for categorical variables. To examine the effect of baseline IL-17A levels on disease activity score 28 (DAS28) ESR response, analysis of covariance (ANCOVA) was used after adjusting for baseline DAS28 ESR, RF positivity, and levels of TNF-*α*, IL-21, and IL-23. A *P* value < 0.05 was considered statistically significant. All statistical analyses were performed using SPSS statistics version 19 (IBM, Chicago, IL, USA) and graphics were generated in GraphPad Prism version 5 (La Jolla, CA, USA).

## 3. Results

### 3.1. Study Population

Clinical characteristics are shown in Table 1. Patients were on average 52.2 years old, 88.6% were females, and RF was positive in 72.7%. Patients had moderate-to-severe RA for ≥6 months with the mean DAS28 ESR score (±SEM) of 6.12 ± 0.12 at baseline. The mean disease duration was 10.9 ± 1.2 years. All patients took MTX with the mean dose of 14.6 mg per week. In addition, 29.5% and 11.4% of patients received hydroxychloroquine and sulfasalazine, respectively. Furthermore, 6.8% received TNF-*α* inhibitors prior to commencement of the study.

### 3.2. Baseline Serum Cytokine Levels and Changes in DAS28 ESR in Patients Who Did and Did Not Achieve Remission at 12 Weeks of TCZ Therapy

**Figure 1:**
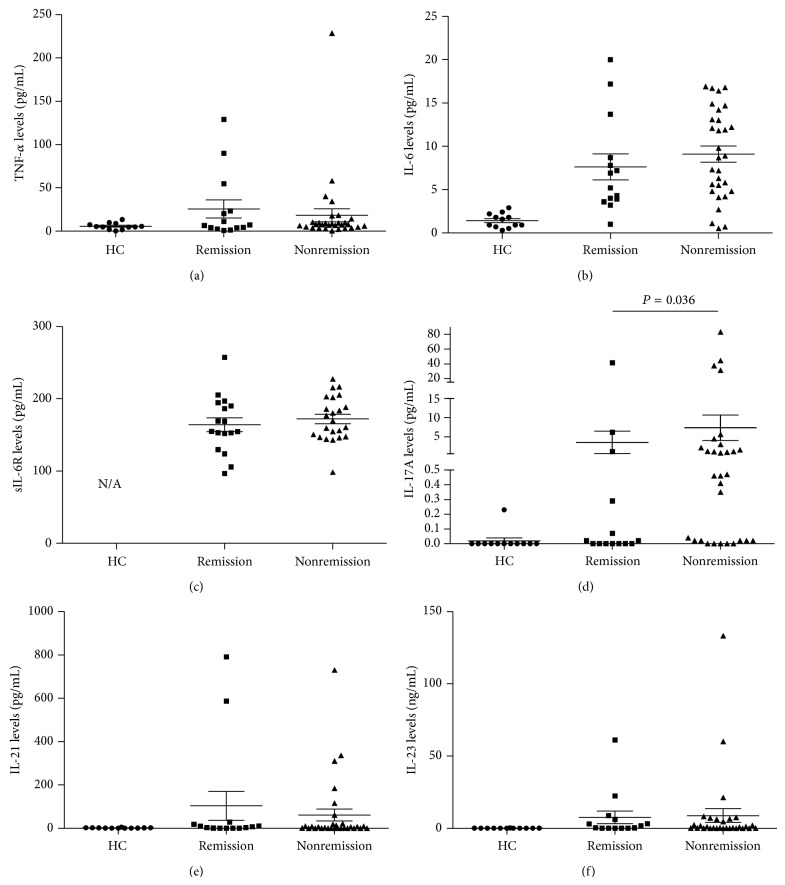
Baseline IL-17A levels are lower in the patients who reached DAS28 ESR remission at 12 weeks of tocilizumab treatment. Baseline serum levels of TNF-*α* (a), IL-6 (b), sIL-6R (c), IL-21 (e), and IL-23 (f) did not differ between the patients with DAS28 ESR remission (*n* = 14) and nonremission (*n* = 30) at 12 weeks of tocilizumab treatment. Only IL-17A levels were significantly lower in the remission patients (d). Of note, sIL-6R levels were not checked in healthy controls. Dot represents each person and bars represent the mean value and SEM. *P* value was assessed by Mann-Whitney* U* test. HC = healthy controls and N/A = not assayed.

Among the 44 patients, 14 (31.8%) patients achieved DAS28 ESR remission at 12 weeks of TCZ therapy and 30 (68.2%) patients did not. At baseline, IL-17A levels were significantly lower in the remission group than in the nonremission group (3.50 ± 2.94 versus 7.34 ± 3.34 pg/mL; *P* < 0.036). Levels of TNF-*α*, IL-6, sIL-6R, IL-21, and IL-23 did not differ between the groups (Figure 1).

### 3.3. Baseline Clinical Characteristics and Serum Cytokines Levels in the IL-17A Low Group and the IL-17A High Group

**Figure 2:**
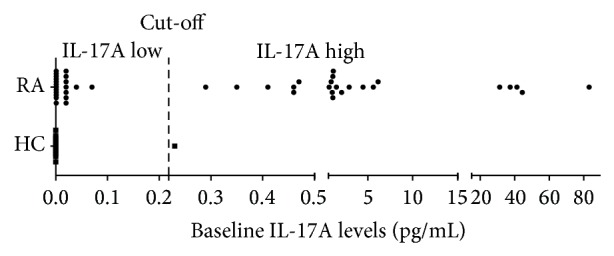
Bimodal distribution of IL-17A levels in the patients. Upper normal value of IL-17A level was defined as the mean serum IL-17A levels + 3SD in healthy controls (*n* = 12). IL-17A levels of RA patients were divided into the IL-17A low (*n* = 21) and high (*n* = 23) groups. Vertical line marks the cut-off value (i.e., upper normal value of IL-17A levels in the healthy controls). RA = rheumatoid arthritis; HC = healthy controls.

In the 12 healthy controls, IL-17A was detected only in one individual at 0.23 pg/mL. In RA patients, baseline IL-17A levels appeared to follow a bimodal distribution (Figure 2). Accordingly, RA patients were divided into an IL-17A low group and an IL-17A high group using the baseline mean + 3 standard deviation (SD) IL-17A level (i.e., 0.22 pg/mL) in the healthy controls as a cut-off value. Baseline clinical characteristics were comparable between the IL-17A low group (*n* = 21) and the IL-17A high group (*n* = 23). Disease duration and activity expressed as DAS28 ESR did not differ between the groups. Only RF positivity was higher in the IL-17A high group (Table 1). At baseline, TNF-*α*, IL-21, and IL-23 levels were significantly higher in the IL-17A high group than in the IL-17A low group (*P* = 0.001, *P* = 0.003, and *P* = 0.002, resp.). Levels of IL-6 and sIL-6R at baseline were not significantly different between the groups (Table 2).

### 3.4. Low Baseline Levels of IL-17A Were Associated with Better Clinical Response to TCZ Treatment

**Figure 3:**
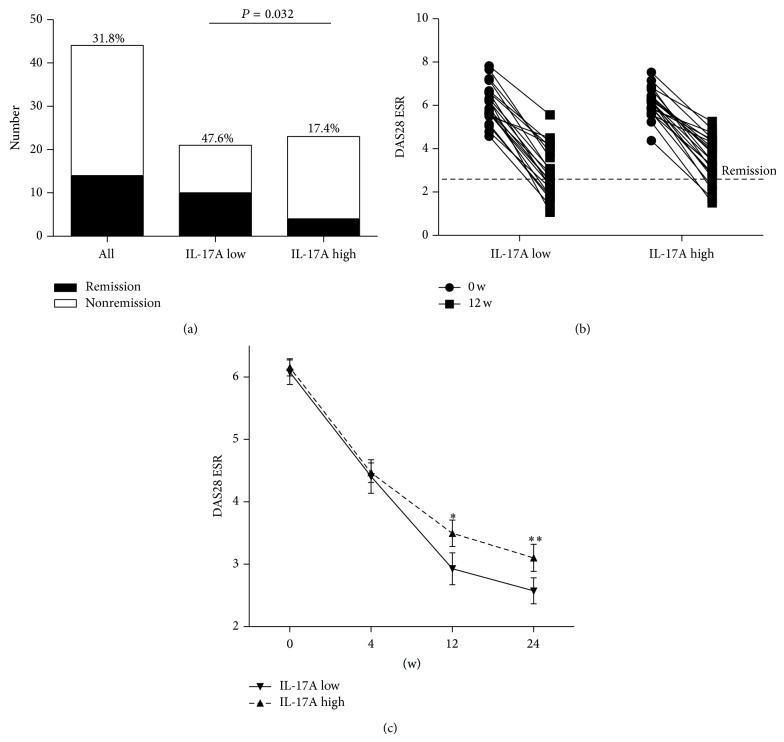
Low baseline levels of IL-17A are associated with DAS28 ESR remission at 12 weeks of tocilizumab treatment. A significantly higher proportion of patients in the IL-17A low group achieved DAS28 remission at 12 weeks as compared to the IL-17A high group (47.6 versus 17.4%, *P* = 0.032) by Fisher's exact test (a). Individual DAS28 ESR improved in both groups. However, the improvement in DAS28 ESR was more profound in the IL-17A low group with higher remission rate (b). The improvement in DAS28 ESR was better in the IL-17A low group at 12 weeks (^∗^ *P* = 0.045) and 24 weeks (^∗∗^ *P* = 0.046) after tocilizumab treatment after adjusting for baseline DAS28 ESR, RF positivity, and levels of TNF-*α*, IL-21, and IL-23. Data represents mean value ± SEM (c).

A significantly higher proportion of patients in the IL-17A low group achieved DAS28 ESR remission at 12 weeks as compared to the IL-17A high group (47.6 versus 17.4%; *P* = 0.032 by Fisher's exact test) (Figure 3(a)). Individual DAS28 ESR scores in each group at baseline and 12 weeks are depicted in Figure 3(b). In the IL-17A low group, the mean DAS28 score improved from 6.07 ± 0.20 at baseline to 2.93 ± 0.25 at 12 weeks, whereas the mean DAS28 score in the IL-17A high group declined from 6.16 ± 0.14 to 3.49 ± 0.21. The improvement in the DAS28 ESR was significantly better in the IL-17A low group compared to the IL-17A high group at 12 weeks (3.15 ± 0.21 versus 2.66 ± 0.17; *P* = 0.045) after adjusting for baseline DAS28 ESR, RF positivity, and levels of TNF-*α*, IL-21, and IL-23. After 12 weeks, 18 (85.7%) of 21 patients in the IL-17A low group and 21 (91.3%) of 23 patients in the IL-17A high group continued the therapy with TCZ over a period of 24 weeks. TCZ was discontinued in five patients due to adverse effects. At 24 weeks, the mean DAS28 ESR score in the IL-17A low and high groups was 2.57 ± 0.21 and 3.10 ± 0.22, respectively. DAS28 ESR improved from baseline significantly more in the IL-17A low group compared to the IL-17A high group at 24 weeks (3.47 ± 0.23 versus 2.99 ± 0.22; *P* = 0.046) as well (Figure 3(c)).

### 3.5. Effect of TCZ on the Levels of Th17-Related Cytokines after Treatment

Levels of IL-6 and IL-17A did not significantly change during treatment. However, levels of IL-21 and IL-23 were decreased significantly at 12 weeks in both DAS28 remission and nonremission patients (all *P* < 0.005). Interestingly, serum levels of TNF-*α* did not change at 12 weeks in the remission group, whereas they increased in the nonremission group. Although baseline sIL-6R levels were not significantly different in both remission and nonremission patients, they significantly increased after TCZ treatment irrespective of the remission status (all *P* < 0.001) (Supplemental Figure  1 in Supplementary Material available online at http://dx.doi.org/10.1155/2015/487230).

## 4. Discussion

To the best of our knowledge, the current study is the first to show the association between the low baseline IL-17A and better clinical response to TCZ treatment in RA patients who had an inadequate response to DMARDs.

In the present study, DAS28 improvement was significantly better in the IL-17A low group than in the IL-17A high group at 12 and 24 weeks of TCZ treatment. Only baseline IL-17A levels differed between patients who did and did not achieve remission, whereas other clinical and laboratory features including baseline sIL-6R levels did not differ. This is in contrast to a previous report that low baseline levels of sIL-6R were associated with better clinical response to TCZ [14]. The difference could be explained in part by the different demographic and clinical features of the studied patients; the patients in the prior study had a lower disease activity (mean DAS28 ESR 5.1) with relatively short disease duration (mean 4.5 years) and received infrequently concomitant MTX (33%) as compared with the patients in our study. As such, it is conceivable that RA progression might be associated with emergence of Th17 cells and that those RA patients with additional IL-17 signature respond less to TCZ, which targets IL-6 signaling. It would be interesting to define whether cytokine profiles change over time as RA progresses. Furthermore, the prior observation that young age (<55 years), baseline CRP > 1 mg/dL, and no history of cerebrovascular disease were associated with European League Against Rheumatism remission at 6 months in RA patients treated with TCZ [15] could not be confirmed in our study.

IL-6 is required for IL-21 production by naïve T cells. Both IL-6 and IL-21 lead to B-cell activation that contributes to RA progression [16]. In addition, IL-21 induces the expression of IL-23R. Subsequently, IL-21 and IL-23 along with TGF-*β* induce expression of ROR*γ*t, which is the transcription factor for Th17 differentiation, and subsequent IL-17 production [17]. As expected, the IL-17A high patients had also increased levels of IL-21 and IL-23 (Table 2), consistent with the prior reports that Th17 are enriched in a subset of RA patients [18, 19]. Therefore, it is conceivable that blockade of the IL-6 signaling by TCZ decreases production of both IL-21 and IL-23 [17].

Interestingly, the levels of TNF-*α* and IL-6 were not associated with better TCZ treatment response (Figure 1). Instead, only low IL-17A levels were associated with better clinical response to TCZ, suggesting that the additional presence of IL-17 or even Th17 cells lead to worse response to IL-6 blockade. Indeed, an incomplete response to TNF-*α* inhibitors was reportedly associated with high baseline IL-17 levels in RA patients [20, 21]. Similarly, patients with type I interferon high signature may not respond to rituximab treatment [22]. Therefore, RA is a heterogeneous disease group encompassing different subsets with varying immunologic abnormalities [23] and patients with elevated IL-17A levels might represent a unique RA subset.

In the present study, significant difference in DAS response between the IL-17A high and low groups might indicate additional contribution of IL-17A to the total inflammatory burden in RA. This might explain the modest treatment effect of secukinumab, anti-IL-17A monoclonal antibody, in treatment of RA [24]. It is tempting to speculate that blockade of IL-17 in addition to IL-6 or TNF-*α* inhibition might improve the clinical response in those RA patients with high baseline IL-17A levels.

## 5. Conclusions

Low baseline serum levels of IL-17A are associated with better clinical response to TCZ in RA patients. A larger prospective study is needed to confirm the predictive value of IL-17A levels in treatment response. Measuring IL-17A levels might help develop a new personalized RA treatment strategy.

## Supplementary Material

Supplemental Figure 1. Changes in serum cytokine levels in DAS28 ESR remission patients and non-remissions at 12 weeks after tocilizumab treatment. The changes in serum levels of TNF-α (A), IL-6 (B), sIL-6R (C), IL-17A (D), IL-21 (E) and IL-23 (F) in 14 DAS28 ESR remission patients and 30 non-remission patients at 12 weeks after tocilizumab treatment (IL-6 and sIL-6R levels were shown respectively at 8 weeks and 24 weeks because we did not measure at 12 weeks). Dot represents each person and bars represent the mean value and SEM. The *p*-value was assessed by Mann-Whitney U test or Wilcoxon's signed rank test.

## Figures and Tables

**Table 1:** Baseline clinical characteristics of the IL-17A low group and the IL-17A high group.

	All (*n* = 44)	IL-17A low (*n* = 21)	IL-17A high (*n* = 23)	*P* value
Age, years	52.2 ± 1.6	50.9 ± 1.9	53.5 ± 2.5	0.285
Female (%)	39 (88.6)	19 (90.5)	20 (87.0)	1.000
Disease duration, years	10.9 ± 1.2	10.8 ± 1.9	11.0 ± 1.5	0.716
RF positivity (%)	32 (72.7)	11 (52.4)	21 (91.3)	0.006
ESR (mm/h)	51.7 ± 3.6	48.4 ± 5.9	54.7 ± 4.4	0.217
CRP (mg/dL)	2.6 ± 0.4	2.5 ± 0.4	2.6 ± 0.6	0.733
DAS28 ESR	6.12 ± 0.12	6.07 ± 0.20	6.16 ± 0.14	0.549
Prednisolone users (%)	36 (81.8)	19 (90.5)	17 (73.9)	0.245
Prednisolone equivalent dose (mg/day)	3.8 ± 0.4	4.2 ± 0.6	3.3 ± 0.6	0.302
Concomitant DMARDs				
MTX users (%)	44 (100)	21 (100)	23 (100)	1.000
MTX (mg/wk)	14.6 ± 0.5	14.4 ± 0.7	14.7 ± 0.6	0.887
HCQ (%)	13 (29.5)	5 (23.8)	8 (34.8)	0.426
SSZ (%)	5 (11.4)	5 (23.8)	0 (0)	0.013
Previous TNF-*α* inhibitors (%)	3 (6.8)	3 (14.3)	0 (0)	0.100

Data are presented as mean (±SEM) for continuous data and number (percentage) for categorical variables. RF = rheumatoid factors; ESR = erythrocyte sedimentation rate; CRP = C-reactive protein; DAS = disease activity score; DMARDs = disease-modifying antirheumatic drugs; MTX = methotrexate; HCQ = hydroxychloroquine; SSZ = sulfasalazine; TNF-*α* = tumor necrosis factor-alpha.

**Table 2:** Comparison of baseline serum cytokine levels in the IL-17A low group and the IL-17A high group.

	IL-17A low (*n* = 21)	IL-17A high (*n* = 23)	*P* value
IL-17A levels	0.01 ± 0.00	11.69 ± 4.43	<0.001
TNF-*α* levels	6.38 ± 1.23	33.84 ± 11.00	0.001
IL-6 levels	8.46 ± 1.14	8.77 ± 1.14	0.879
sIL-6R levels	162.94 ± 7.68	170.37 ± 7.09	0.672
IL-21 levels	4.34 ± 1.48	140.05 ± 50.97	0.003
IL-23 levels	1.23 ± 0.54	15.11 ± 6.45	0.002

Data are presented as mean (±SEM) for continuous variables. TNF-*α* = tumor necrosis factor-alpha; IL = interleukin; sIL-6R = soluble interleukin 6 receptor.

## References

[1] Harris E. D. (1990). Rheumatoid arthritis. Pathophysiology and implications for therapy. *The New England Journal of Medicine*.

[2] Feldmann M., Maini R. N. (2003). Lasker clinical medical research award. TNF defined as a therapeutic target for rheumatoid arthritis and other autoimmune diseases. *Nature Medicine*.

[3] Naka T., Nishimoto N., Kishimoto T. (2002). The paradigm of IL-6: from basic science to medicine. *Arthritis Research*.

[4] Tanaka T., Kishimoto T. (2012). Targeting interleukin-6: all the way to treat autoimmune and inflammatory diseases. *International Journal of Biological Sciences*.

[5] Mihara M., Ohsugi Y., Kishimoto T. (2009). Evidence for the role of Th17 cell inhibition in the prevention of autoimmune diseases by antiinterluekin-6 receptor antibody. *BioFactors*.

[6] van Hamburg J. P., Asmawidjaja P. S., Davelaar N. (2011). Th17 cells, but not Th1 cells, from patients with early rheumatoid arthritis are potent inducers of matrix metalloproteinases and proinflammatory cytokines upon synovial fibroblast interaction, including autocrine interleukin-17A production. *Arthritis and Rheumatism*.

[7] Bettelli E., Carrier Y., Gao W. (2006). Reciprocal developmental pathways for the generation of pathogenic effector T_H_17 and regulatory T cells. *Nature*.

[8] Burmester G. R., Feist E., Kellner H., Braun J., Iking-Konert C., Rubbert-Roth A. (2011). Effectiveness and safety of the interleukin 6-receptor antagonist tocilizumab after 4 and 24 weeks in patients with active rheumatoid arthritis: the first phase IIIb real-life study (TAMARA). *Annals of the Rheumatic Diseases*.

[9] Genovese M. C., McKay J. D., Nasonov E. L. (2008). Interleukin-6 receptor inhibition with tocilizumab reduces disease activity in rheumatoid arthritis with inadequate response to disease-modifying antirheumatic drugs: the tocilizumab in combination with traditional disease-modifying antirheumatic drug therapy study. *Arthritis and Rheumatism*.

[10] Maini R. N., Taylor P. C., Szechinski J. (2006). Double-blind randomized controlled clinical trial of the interleukin-6 receptor antagonist, tocilizumab, in European patients with rheumatoid arthritis who had an incomplete response to methotrexate. *Arthritis and Rheumatism*.

[11] Nishimoto N., Miyasaka N., Yamamoto K., Kawai S., Takeuchi T., Azuma J. (2009). Long-term safety and efficacy of tocilizumab, an anti-IL-6 receptor monoclonal antibody, in monotherapy, in patients with rheumatoid arthritis (the STREAM study): evidence of safety and efficacy in a 5-year extension study. *Annals of the Rheumatic Diseases*.

[12] Smolen J. S., Beaulieu A., Rubbert-Roth A. (2008). Effect of interleukin-6 receptor inhibition with tocilizumab in patients with rheumatoid arthritis (OPTION study): a double-blind, placebo-controlled, randomised trial. *The Lancet*.

[13] Shimamoto K., Ito T., Ozaki Y. (2013). Serum interleukin 6 before and after therapy with tocilizumab is a principal biomarker in patients with rheumatoid arthritis. *The Journal of Rheumatology*.

[14] Nishina N., Kikuchi J., Hashizume M., Yoshimoto K., Kameda H., Takeuchi T. (2014). Baseline levels of soluble interleukin-6 receptor predict clinical remission in patients with rheumatoid arthritis treated with tocilizumab: implications for molecular targeted therapy. *Annals of the Rheumatic Diseases*.

[15] Pers Y.-M., Fortunet C., Constant E. (2014). Predictors of response and remission in a large cohort of rheumatoid arthritis patients treated with tocilizumab in clinical practice. *Rheumatology*.

[16] Carbone G., Wilson A., Diehl S. A., Bunn J., Cooper S. M., Rincon M. (2013). Interleukin-6 receptor blockade selectively reduces IL-21 production by CD4 T cells and IgG4 autoantibodies in rheumatoid arthritis. *International Journal of Biological Sciences*.

[17] Zhou L., Ivanov I. I., Spolski R. (2007). IL-6 programs T_H_-17 cell differentiation by promoting sequential engagement of the IL-21 and IL-23 pathways. *Nature Immunology*.

[18] Kim J., Kang S., Kim J., Kwon G., Koo S. (2013). Elevated levels of T helper 17 cells are associated with disease activity in patients with rheumatoid arthritis. *Annals of Laboratory Medicine*.

[19] Miao J., Zhang K., Lv M. (2014). Circulating Th17 and Th1 cells expressing CD161 are associated with disease activity in rheumatoid arthritis. *Scandinavian Journal of Rheumatology*.

[20] Alzabin S., Abraham S. M., Taher T. E. (2012). Incomplete response of inflammatory arthritis to TNF*α* blockade is associated with the Th17 pathway. *Annals of the Rheumatic Diseases*.

[21] Chen D.-Y., Chen Y.-M., Chen H.-H., Hsieh C.-W., Lin C.-C., Lan J.-L. (2011). Increasing levels of circulating Th17 cells and interleukin-17 in rheumatoid arthritis patients with an inadequate response to anti-TNF-*α* therapy. *Arthritis Research & Therapy*.

[22] Thurlings R. M., Boumans M., Tekstra J. (2010). Relationship between the type I interferon signature and the response to rituximab in rheumatoid arthritis patients. *Arthritis and Rheumatism*.

[23] Park J. K. (2011). Rheumatoid arthritis subgroup with type I interferon signature: comment on the article by Thurlings et al. *Arthritis and Rheumatism*.

[24] Genovese M. C., Durez P., Richards H. B. (2013). Efficacy and safety of secukinumab in patients with rheumatoid arthritis: a phase II, dose-finding, double-blind, randomised, placebo controlled study. *Annals of the Rheumatic Diseases*.

