# Erratum to “Low Baseline Interleukin-17A Levels Are Associated with Better Treatment Response at 12 Weeks to Tocilizumab Therapy in Rheumatoid Arthritis Patients”

**DOI:** 10.1155/2017/7869412

**Published:** 2017-08-20

**Authors:** Sang Jin Lee, Won Park, Sung Hwan Park, Seung-Cheol Shim, Han Joo Baek, Dae-Hyun Yoo, Hyun Ah Kim, Soo Kon Lee, Yun Jong Lee, Young Eun Park, Hoon-Suk Cha, Jin Kyun Park, Eun Young Lee, Eun Bong Lee, Yeong Wook Song

**Affiliations:** ^1^Division of Rheumatology, Seoul National University Hospital, Seoul, Republic of Korea; ^2^Department of Molecular Medicine and Biopharmaceutical Sciences, Graduate School of Convergence Science and Technology and College of Medicine, Medical Research Institute, Seoul National University, Seoul, Republic of Korea; ^3^Division of Rheumatology, Inha University Hospital, Incheon, Republic of Korea; ^4^Division of Rheumatology, The Catholic University of Korea Seoul St. Mary's Hospital, Seoul, Republic of Korea; ^5^Division of Rheumatology, Eulji University Hospital, Daejeon, Republic of Korea; ^6^Division of Rheumatology, Gachon University Gil Medical Center, Incheon, Republic of Korea; ^7^Division of Rheumatology, Hanyang University Medical Center, Seoul, Republic of Korea; ^8^Division of Rheumatology, Hallym University Medical Center, Anyang, Republic of Korea; ^9^Division of Rheumatology, Yonsei University Health System, Seoul, Republic of Korea; ^10^Division of Rheumatology, Seoul National University Bundang Hospital, Seongnam, Republic of Korea; ^11^Division of Rheumatology, Pusan National University Hospital, Busan, Republic of Korea; ^12^Division of Rheumatology, Samsung Medical Center, Seoul, Republic of Korea

In the article titled “Low Baseline Interleukin-17A Levels Are Associated with Better Treatment Response at 12 Weeks to Tocilizumab Therapy in Rheumatoid Arthritis Patients” [1], the name of the ninth author was given incorrectly as Yun Jong Leee. The author's name should have been written as Yun Jong Lee. The revised authors' list is shown above.
