# Supplementary figures and images for: Low Baseline Interleukin-17A Levels Are Associated with Better Treatment Response at 12 Weeks to Tocilizumab Therapy in Rheumatoid Arthritis Patients
Source: J Immunol Res. 2015 Apr 2;2015:487230. doi: 10.1155/2015/487230 (PMC4398953; doi:10.1155/2015/487230)

Supplemental Figure 1

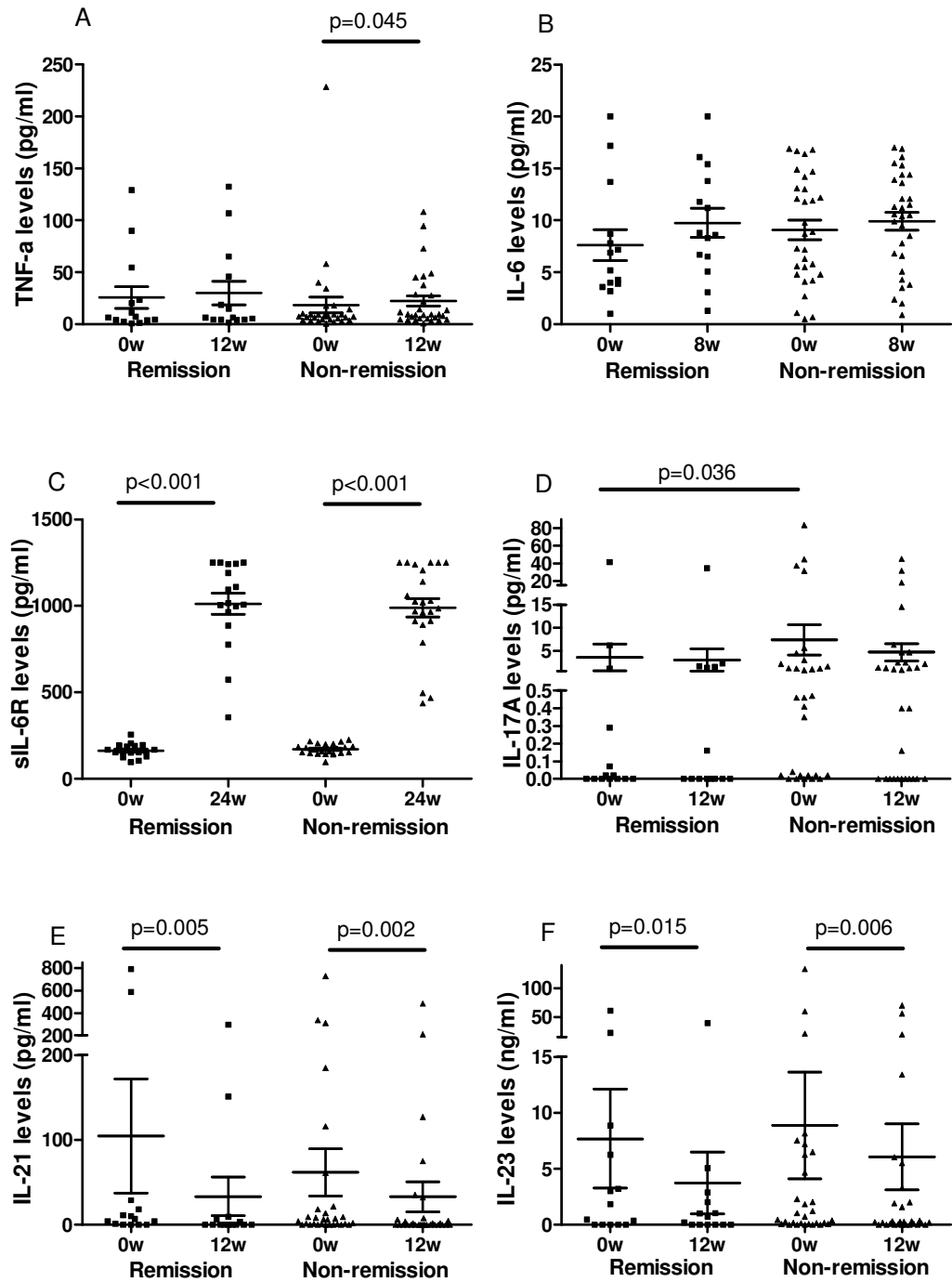

Supplement: Supplementary file 1 — Supplemental Figure 1. Changes in serum cytokine levels in DAS28 ESR remission patients and non-remissions at 12 weeks after tocilizumab treatment. The changes in serum levels of TNF-α (A), IL-6 (B), sIL-6R (C), IL-17A (D), IL-21 (E) and IL-23 (F) in 14 DAS28 ESR remission patients and 30 non-remission patients at 12 weeks after tocilizumab treatment (IL-6 and sIL-6R levels were shown respectively at 8 weeks and 24 weeks because we did not measure at 12 weeks). Dot represents each person and bars represent the mean value and SEM. The p-value was assessed by Mann-Whitney U test or Wilcoxon's signed rank test. [file 487230.f1.pdf]
